# Toll-like receptor 2 -196 to -174 del polymorphism influences the susceptibility of Han Chinese people to Alzheimer's disease

**DOI:** 10.1186/1742-2094-8-136

**Published:** 2011-10-11

**Authors:** Jin-Tai Yu, Shan-Mao Mou, Li-Zhu Wang, Cai-Xia Mao, Lan Tan

**Affiliations:** 1 Department of Neurology, Qingdao Municipal Hospital, School of Medicine, Qingdao University, No.5 Donghai Middle Road, Qingdao 266071, China; 2 College of Medicine and Pharmaceutics, Ocean University of China, Qingdao 266003, China; 3 Taishan Medical University, Taian, Shandong Province 271016, China; 4 Department of VIP, Affiliated Hospital of Nantong University, Nantong, Jiangsu Province 226001, China

**Keywords:** Alzheimer's disease, toll-like receptor 2, polymorphism, association study

## Abstract

**Background:**

Toll-like receptor 2 (*TLR2*) represents a reasonable functional and positional candidate gene for Alzheimer's disease (AD) as it is located under the linkage region of AD on chromosome 4q, and functionally is involved in the microglia-mediated inflammatory response and amyloid-β clearance. The -196 to -174 del polymorphism affects the *TLR2 *gene and alters its promoter activity.

**Methods:**

We recruited 800 unrelated Northern Han Chinese individuals comprising 400 late-onset AD (LOAD) patients and 400 healthy controls matched for gender and age. The -196 to -174 del polymorphism in the *TLR2 *gene was genotyped using the polymerase chain reaction (PCR) method.

**Results:**

There were significant differences in genotype (P = 0.026) and allele (P = 0.009) frequencies of the -196 to -174 del polymorphism between LOAD patients and controls. The del allele was associated with an increased risk of LOAD (OR = 1.31, 95% CI = 1.07-1.60, Power = 84.9%). When these data were stratified by apolipoprotein E (*ApoE*) ε4 status, the observed association was confined to *ApoE *ε4 non-carriers. Logistic regression analysis suggested an association of LOAD with the polymorphism in a recessive model (OR = 1.64, 95% CI = 1.13-2.39, Bonferroni corrected P = 0.03).

**Conclusions:**

Our data suggest that the -196 to -174 del/del genotype of *TLR2 *may increase risk of LOAD in a Northern Han Chinese population.

## Background

Toll-like receptor 2 (*TLR2*) represents a reasonable functional and positional candidate gene for Alzheimer's disease (AD) as it is located under the linkage region of AD on chromosome 4q [1], and is functionally involved in the microglia-mediated inflammatory response and amyloid β (Aβ) clearance [2-6]. Genetic studies on the *TLR2 *gene have identified a number of polymorphisms which have been shown to affect host defense, disease progression and be linked to differential disease susceptibilities [7]. We have assessed the involvement of 7 single nucleotide polymorphisms (SNPs) (Arg 677Trp, Arg753Gln, rs1898830, rs11938228, rs3804099, rs3804100, and rs7656411) as well as a short tandem GT repeat polymorphism in intron 2 of the *TLR2 *gene in the risk of developing late-onset AD (LOAD) [8,9], and found an association of GT repeat polymorphism with an increased risk for LOAD in our previous studies. A 22-bp nucleotide deletion at position -196 to -174 of the untranslated 5'-region in *TLR2 *gene is associated with reduced transcriptional activity compared to the wild type allele in luciferase reporter assays [10]. This polymorphism has already been shown to be associated with an increased risk of noncardiac gastric cancer, susceptibility to cervical cancer, hepatitis C viral loads and susceptibility to hepatocellular carcinoma [11-13]. In light of the important role that TLR2 plays with respect to the immune response in the pathogenesis of AD [2-6], we hypothesized that the -196 to -174 del/ins polymorphism in the *TLR2 *gene might be associated with AD.

## Methods

### Subjects

Our study was comprised of 400 sporadic LOAD cases (189 female and 211 male; age > 65 years; mean age = 82.8 ± 7.1 years; age at onset = 75.4 ± 5.9 years) and 400 healthy controls subjects matched for sex and age (189 female and 211 male; mean age = 81.4 ± 5.4 years). All participants originated from Northern Han Chinese populations. A clinical diagnosis of probable AD was established according to the criteria of National Institute of Neurological and Communicative Disorders and Stroke and the Alzheimer's disease and Related Disorders Association (NINCDS-ADRDA) [14]. All AD cases were defined as sporadic because family histories showed no mention of first-degree relatives with dementia. Control subjects were unrelated individuals selected from the Health Examination Center of the Qingdao Municipal Hospital. These subjects were confirmed healthy and neurologically normal by complete neurological and medical examinations comprised of medical history and laboratory examinations. Written informed consent was obtained from all individuals and the study was approved by the Institute Ethical Committee.

### Genotyping

Genomic DNA was extracted from peripheral blood leukocytes using the standard method [15]. Polymorphisms at *TLR2 *-196 to -174del were investigated using the polymerase chain reaction (PCR) method, following the procedures described by Tahara et al [11]. The apolipoprotein E (*ApoE*) genotype was determined according to the method described by Donohoe et al [16]. Two investigators independently reviewed all results.

### Statistical analysis

Hardy-Weinberg equilibrium was assessed using the Chi-square test. Genotype and allele distributions were compared using the Chi-square test. Differences in allele and genotype distribution between cases and controls were analyzed using logistic regression adjusted for age and *ApoE *ε4 status under various genetic models. The Wald P value was multiplied by 3 as a Bonferroni adjustment for the 3 genetic models tested. The P value, odds ratios (OR) and 95% confidence intervals (CI) were calculated. Estimation of the statistical power was performed with the STPLAN 4.3 software. Data were analyzed using a commercially available statistical package (SPSS Version 13.0, SPSS Inc., Chicago, IL). The criterion used for significant differences is P < 0.05.

## Results

The alleles and genotypes frequencies of LOAD patients and controls in the total sample and after stratification for *ApoE *ε4 allele are given in Table 1. The distribution of genotypes of *TLR2 *polymorphisms were within the range of Hardy-Weinberg equilibrium (P = 0.21). There were significant differences in genotype and allele frequencies between LOAD and control groups (genotype P = 0.026, allele P = 0.009). The -196 to -174 del allele significantly raised the risk of developing LOAD (OR = 1.31, 95%CI = 1.07-1.60, Power = 84.9%). In subjects without *ApoE *ε4 allele, the allele and genotype distributions between LOAD patients and controls remain significantly different (genotype P = 0.027, allele P = 0.009). However, in subjects with the *ApoE *ε4 allele, there were no significant differences. In order to rule out confounding in our crude association analyses, we reevaluated the polymorphism effect under 3 different models using logistic regression adjusting for age and *ApoE *ε4 status (Table 2). The -196 to -174 del polymorphism was still found to increase the risk of LOAD via a recessive model (OR = 1.64, 95% CI = 1.13-2.39, P = 0.01, Bonferroni corrected P = 0.03).

**Table 1:** Distribution of the *TLR2 *-196 to -174 del polymorphism in LOAD cases and controls.

	N	Genotypes n (%)				Alleles n (%)		
		**ins/ins**	**del/ins**	**del/del**	**P**	**del**	**ins**	**P**
		
AD	400	150(37.5)	161(40.2)	89(22.2)	0.026	461(57.6)	339(42.4)	0.009
Controls	400	172(43.0)	168(42.0)	60(15.0)		512(64.0)	288(36.0)	
*ApoE*ε4(-)								
AD	260	94(36.2)	104(40.0)	62(23.8)	0.027	292(56.2)	228(43.8)	0.009
Controls	339	144(42.5)	143(42.2)	52(15.3)		431(63.6)	247(36.4)	
*ApoE*ε4(+)								
AD	140	56(40.0)	57(40.7)	27(19.3)	0.526	169(60.4)	111(39.6)	0.251
Controls	61	28(45.9)	25(41.0)	8(13.1)		81(66.4)	41(33.6)	

*ApoE *ε4 (+): subjects who carry one or two ε4 alleles; *ApoE *ε4 (-): subjects who do not carry an ε4 allele.

**Table 2:** Logistic regression analysis of the -196 to -174 del polymorphism within *TLR2*.

Model	OR (95%CI)	Wald	P	Pc
Dom	1.172(0.776-1.770)	0.568	0.451	NC
Rec	1.642(1.127-2.392)	6.669	0.010	0.030
Add	1.279(1.010-1.620)	4.179	0.041	0.123

Adjusted for age and for carriage of at least one *ApoE *ε4 allele.

Dom, dominant model: 1 (del/del+del/ins) versus 0 (ins/ins); Rec, recessive model: 1 (del/del) versus 0 (del/ins + ins/ins); Add, additive model: 0 (ins/ins) versus 1 (del/ins) versus 2 (del/del); OR, odds ratio; CI, confidence interval. Pc, corrected P for multiple testing by Bonferroni correction (P value was multiplied by 3 as a Bonferroni adjustment for the 3 genetic models tested). NC, not calculated.

## Discussion

Many experimental and clinical studies have suggested that TLR2 might play an important role in the pathogenesis of AD [2-6]. TLR2 is a member of pattern-recognition receptors in the innate immune system [7]. Increased levels of *TLR2 *mRNA have been found in microglia isolated from AD patients [2]. Jana et al. have supplied several lines of evidence supporting the opinion that Aβ peptides activate microglia via TLR2 as inhibition of *TLR2 *through function-blocking antibodies or siRNA knockdown prevents fibrillar forms of Aβ from inducing nitrite, interleukin-6 (IL-6), or tumor necrosis factor-alpha (TNF-α) production [3]. Although an increasing volume of data favors TLR2 -mediated neurotoxicity, TLR2 may also be essential for Aβ clearance and in that way provide neuroprotection in AD [4]. Reed-Geaghan et al. reported that CD14, TLR4, and TLR2 are necessary for binding fibrillar Aβ (fAβ) to the cell surface, and are required for phenotypic activation of microglia and induction of phagocytosis [5]. Richard et al. demonstrated that *TLR2 *deficiency in transgenic AD mice could increase Aβ deposition and accelerate cognitive decline [6].

The -196 to -174 del polymorphism in the *TLR2 *gene, located on chromosome 4, causes a 22 bp nucleotide deletion that alters the promoter activity of *TLR2*. The *TLR2 *del/del genotype is reported to show decreased transactivation of responsive promoters [10]. Consequently, it might be speculated that expression of *TLR2 *in microglial cells might exhibit low levels with the del/del genotype. Further, it can be presumed that the del/del genotype is more conducive to the occurrence of AD. Our results suggest a significant association between the -196 to -174 del allele of *TLR2 *and the risk of developing LOAD in the Han Chinese population. Interestingly, this association was restricted to non-*ApoE *ε4 carriers, as no association was found for *ApoE *ε4 carriers. One possible interpretation is that the genetic effect of *TLR2 *is relevant in predisposing to AD only in the absence of the *ApoE *ε4 allele, while in ε4 carriers the genetic effect is determined by this strong susceptibility factor. There is overlap in the study populations used in present study and in our previous study [9]. Linkage disequilibrium (LD) between the GT repeat polymorphism and the -196 to -174 del polymorphism within the *TLR2 *gene in the overlap study population was measured by calculating the D' and r2 statistics. These were found to be in weak linkage disequilibrium (D' = 0.376 and r^2 ^= 0.009). Hence, haplotype frequencies were not estimated, and both the GT repeat and the -196 to -174 del polymorphisms might independently influence the risk of LOAD.

## Conclusions

Our data suggest that the -196 to -174 del/del genotype of *TLR2 *may increase the risk of LOAD in a Northern Han Chinese population. Additional independent replications and functional genetic analyses are warranted to elucidate the potential mechanisms and the epidemiologic relevance of these associations.

## Abbreviations

Aβ: amyloid β; AD: Alzheimer's disease; ApoE: apolipoprotein E; CI: confidence intervals; IL-6: interleukin-6; LD: linkage disequilibrium; LOAD: late-onset AD; NINCDS-ADRDA: National Institute of Neurological and Communicative Disorders and Stroke and the Alzheimer's disease and Related Disorders Association; ORs: odds ratios; PCR: polymerase chain reaction; SNPs: single nucleotide polymorphisms; TNF-α: tumor necrosis factor-alpha; TLR2: toll-like receptor 2; TLRs: toll-like receptors; fAβ: fibrillar Aβ.

## Competing interests

The authors declare that they have no competing interests.

## Authors' contributions

LT and JTY contributed to the design of the study. JTY, SMM, LZW, CXM and LT were involved in sample acquisition and processing. JTY, SMM, and LZW performed the statistical analyses. JTY and LT drafted the manuscript and contributed to its final version. All authors read and approved the final manuscript.
